# The scope of costs in alcohol studies: Cost-of-illness studies differ from economic evaluations

**DOI:** 10.1186/1478-7547-8-15

**Published:** 2010-07-06

**Authors:** Paul F van Gils, Heleen H Hamberg-van Reenen, Matthijs van den Berg, Luqman Tariq, G Ardine de Wit

**Affiliations:** 1 National Institute for Public Health and the Environment, Centre for Prevention and Health Services Research, Bilthoven, The Netherlands; 2 National Institute for Public Health and the Environment, Centre for Public Health Forecasting, Bilthoven, The Netherlands; 3 Julius Centre for Health Sciences and Primary Care, University Medical Centre Utrecht, The Netherlands

## Abstract

**Background:**

Alcohol abuse results in problems on various levels in society. In terms of health, alcohol abuse is not only an important risk factor for chronic disease, but it is also related to injuries. Social harms which can be related to drinking include interpersonal problems, work problems, violent and other crimes. The scope of societal costs related to alcohol abuse in principle should be the same for both economic evaluations and cost-of-illness studies. In general, economic evaluations report a small part of all societal costs. To determine the cost- effectiveness of an intervention it is necessary that all costs and benefits are included. The purpose of this study is to describe and quantify the difference in societal costs incorporated in economic evaluations and cost-of-illness studies on alcohol abuse.

**Method:**

To investigate the economic costs attributable to alcohol in cost-of-illness studies we used the results of a recent systematic review (June 2009). We performed a PubMed search to identify economic evaluations on alcohol interventions. Only economic evaluations in which two or more interventions were compared from a societal perspective were included. The proportion of health care costs and the proportion of societal costs were estimated in both type of studies.

**Results:**

The proportion of healthcare costs in cost-of-illness studies was 17% and the proportion of societal costs 83%. In economic evaluations, the proportion of healthcare costs was 57%, and the proportion of societal costs was 43%.

**Conclusions:**

The costs included in economic evaluations performed from a societal perspective do not correspond with those included in cost-of-illness studies. Economic evaluations on alcohol abuse underreport true societal cost of alcohol abuse. When considering implementation of alcohol abuse interventions, policy makers should take into account that economic evaluations from the societal perspective might underestimate the total effects and costs of interventions.

## Introduction

Alcohol abuse results in problems on various levels in society. In terms of health, alcohol abuse is not only an important risk factor for chronic disease, but it is also related to unintentional and intentional injuries [1-3]. On the social level the WHO Expert Committee on Problems Related to Alcohol Consumption reported that social harms which can be related to drinking include interpersonal problems, work problems, violent and other crimes [2].

From the economic point of view, the estimated tangible costs of alcohol in the European Union were €125 billion in 2003, including €59 billion worth of lost productivity through absenteeism, unemployment and lost working years due to premature death [4]. Another study reported that the weighted average costs in four high-income countries (France, USA, Scotland and Canada) were 1.4% of the gross domestic product [3].

To reduce the negative effect of alcohol abuse it is necessary for countries to develop an alcohol policy and implement prevention programs. An alcohol policy can be defined as a set of measures in a jurisdiction or society aimed at minimizing the health and social problems from alcohol consumption [2]. The alcohol abuse prevention programs as developed, focus on one of more of the strategies of the WHO: reducing the availability of alcohol, alcohol prices and taxes, restricting the sale of alcohol, restrictions on alcohol marketing, drink-driving countermeasures, education and persuasion [2,5].

Before an intervention may be implemented, a firm evidence base for its effectiveness is needed. The effects of an intervention should be both statistically significant and clinically relevant.

In light of increasing health care costs and limited resources, interventions aimed at reducing alcohol abuse should not only be effective, but efficient or cost-effective too. Nowadays, policy makers require information about the effectiveness of an intervention in relation to its costs. The perspective of an economic evaluation of an alcohol abuse intervention should be taken into account in the decision making process. If a study is performed from a health care perspective only costs are included which are related to health care use. A study performed from a social perspective includes both healthcare costs and all relevant societal costs. In the case of an alcohol abuse intervention performed from the societal perspective, costs due to productivity losses, crime and law enforcement costs also have to be included, in addition to health care costs. Taking into account the public interest, it is important for a policy maker that all costs related to excessive alcohol use are included in an economic evaluation. In other words: to be able to make a clear decision whether getting good value for the money when introducing a preventive intervention, a policymaker needs economic evaluations that include all related societal costs.

Cost-of illness studies which are performed according to the international guidelines of the WHO report all type of healthcare cost and societal costs comprehensively [6]. From economic evaluations from the societal perspective, it is not known whether all types of societal costs are taken into account. To determine the cost- effectiveness of an intervention it is necessary that all costs and benefits are included. The purpose of this study is to describe the cost components and to quantify the difference in societal costs incorporated in economic evaluations and cost-of-illness studies on alcohol abuse. For the comparison of costs incorporated in economic evaluations and in cost-of-illness studies, we do not focus on marginal cost differences between interventions, but rather concentrate on included pre-intervention costs in economic evaluations only.

## Method

A recently published review summarizes the costs attributable to alcohol abuse as involved in cost-of illness studies [3]. One of the purposes of that review was to analyze the full societal effect of alcohol, since cost-of-illness studies are not restricted to health but usually include criminal outcomes and other social detriments. We selected four of the 29 identified cost-of-illness studies. Those studies were methodologically most comparable i.e. those estimating the gross costs and using the same discount rate. Those were studies of high income countries (France, US, Scotland and Canada). We calculated the per-study (unweighted) average proportion of health care costs and societal costs of those four cost-of-illness studies [3]. The types of costs involved in those studies are: total healthcare costs, prevention and research costs, public order and safety costs, criminal damage costs, drink-driving costs, work-related costs, the loss of revenues from compulsory taxes, property damage and premature mortality in the non working population. For the per-study average proportion of healthcare costs, we counted total health care costs and prevention and research costs. To have an estimate of the total societal costs, we counted all other type of costs.

To identify economic evaluations on alcohol interventions, we performed a PubMed search. The search contained the terms: alcohol abuse, alcohol misuse, economic evaluation, cost-effectiveness, cost-utility, life year gained, QALY, prevention, preventive intervention and societal perspective. We searched for economic evaluations that were reported between 2000 and 2009 in English. We only included full economic evaluations performed from the societal perspective in which two or more interventions on alcohol abuse were compared with each other or with usual care [7]. Of the included studies the per-study (unweighted) average proportion of healthcare cost and societal costs at baseline were calculated for the intervention groups. The proportion of the health care costs and the proportion of the societal costs were compared in both cost-of-illness studies and economic evaluations.

To be able to compare the outcomes of cost-of-illness studies and economic evaluations with different base years and different currency units, all local currencies were first transferred to the euro currency values of that time, following the advice of the Organization for Economic Co-operation and Development, and then recalculated to 2008, the year that was chosen as base year for the current study [8,9].

## Results

### Cost-of-illness studies

Table 1 shows the results of the cost-of-illness studies. Differences in types of societal costs that were included in the four investigated cost-of-illness studies were observed. All studies included public order and safety costs and work-related (productivity) costs, and all but one study did include drink-driving costs [10,12]. Productivity costs were estimated using the 'human capital' approach. Other societal types of costs were only reported in one cost-of-illness study [13].

**Table 1:** The costs as reported in four included cost-of-illness studies (recalculated into million 2008€) and the proportion Health Care costs and Societal costs (%)

Study	Healthcare costs	Prevention and research costs	Total Health Care costs	Proportion Health care costs (%)	Public order and safety costs	Criminal damage costs	Drink-driving costs	Work-related costs	Cost of the losses in compulsory taxes	Total Societal costs	Proportion societal costs	Total costs
**Fenoglio (France)**	3,500	698	4,198	19	70		4,393	10,936	2,333.	17,732	81	21,930
**Harwood (USA)**	28,074	1,860	29,934	15	779	8,966	17,948	141,497		164,811	85	198,345
**Varney (Scotland)**	155	3	158	9	142	442		1025		1,608	91	1,766
**Rehm (Canada)**	2,678	110	2,788	24	2,488	127	613	5,772		9,001	76	11,789

Averaged over all the cost-of-illness studies, the proportion of the healthcare costs was 17% of the total cost due to alcohol abuse. The societal costs attributed to 83% of the total alcohol abuse costs.

### Economic evaluations

Our literature search on economic evaluations of preventive interventions directed at alcohol abuse conducted in July 2009, identified 116 potentially relevant studies. After having read the abstracts, we selected 34 articles to read in full, of those we included 5 studies because these studies satisfied our inclusion criterion [14,18]. Reasons for excluding 29 studies were that those were not performed from a societal perspective or were not full economic evaluations. One of included studies was a modeling study [17], the other four were original research. Two of the studies used the QALY as an outcome measure [17,18], while the other studies presented the outcome in surrogate endpoints: reduction in alcohol consumption [14,15] or number of successfully treated persons [16].

Table 2 shows the types of costs involved in the economic evaluations and the absolute cost figures. Healthcare costs were reported in all included economic evaluations, and prevention and research costs were included in four of five economic evaluations [14,17]. Focusing on societal costs, there were large differences in types of costs that were included. Four studies included public order and safety costs [14,15,17,18], and two studies included work related costs, using the 'human capitol' approach. There were no studies that included drink-driving costs and costs of losses in compulsory taxes. We only report the cost components and the absolute costs before the start of an intervention and do not pay attention to incremental or marginal costs.

**Table 2:** The types of baseline costs as reported in five economic evaluations (2008€) and the proportion Health Care costs and Societal costs (%)

Study		Healthcare costs	Prevention and research costs	Total Heath Care costs	Proportion Health Care costs (%)	Public order and safety costs	Criminal damage costs	Drink-driving costs	Work-related costs	Cost of the losses in compulsory taxes	Total societal cost	Proportion Societal costs (%)	Total costs
**Barrett**		4,523		4,523	53	3,146	665		153		3,964	47	8,487
**Parrott**^**1**^	Intervention 1	1,087	74	1,161	35	69	2,099				2,168	65	3,329
	Intervention 2	2,367	627	2,994	60	274	1,759				2,033	40	5,027
**Rychlik**		1,666	10	1,676	77				488		488	23	2,164
**Solberg**		2,418		2,418	33	5,007					5,007	67	7,425
**UKATT**^**1**^	Intervention 1	2,678		2,678	74	83	856				939	26	3,617
	Intervention 2	2,991		2,991	70	144	1130				1,274	30	4,265

Averaged over all economic evaluations the proportion of the healthcare costs at baseline was 57% of the total cost, and of the societal costs amounted for 43%.

## Discussion

Our study shows that the scope of societal costs included in five economic evaluations with a societal perspective was smaller than the scope of societal costs as incorporated in four cost-of-illness studies. We found only 5 full-economic evaluation studies on preventive interventions of alcohol abuse. None of the economic evaluations included all types of societal costs that were represented in the cost-of-illness studies. This means that, although it is claimed that the societal perspective is taken, the societal costs as reported in economic evaluations are incomplete in general. In relation to alcohol abuse it is remarkable that none of the studies included drink-driving costs. Only two studies included work-related costs due to productivity losses. These results corresponds with the findings of Barbosa et al. that a societal perspective has never been taken into full account in economic evaluations [19]. The four cost-of-illness studies used to identify the healthcare and societal costs of alcohol abuse present those costs clearly in general. The only exception is that Fenoglio et al. did not include criminal damage [13].

Averaged over all economic evaluations, the proportion of healthcare costs (57%) and societal cost (43%) does not correspond with the proportion as reported in cost-of-illness studies (17% and 83%, respectively). So, the incremental cost-effectiveness ratios (ICER) that are reported in economic evaluations might be underestimations of the true societal costs and effects of preventive interventions directed at alcohol abuse.

It is known that economic evaluations of alcohol abuse interventions performed from a healthcare perspective underestimate true costs, because only healthcare costs are involved in this kind of studies. It will be useful to policy makers if studies make clear how a life-style intervention directed towards alcohol abuse affects both the health care costs and the societal costs [20]. Studies that claim to be done from a societal perspective show an underestimation too. The real part of societal costs will be much higher than presented in those economic evaluations.

Comparing productivity costs between studies is hampered by the existence of two 'schools' that address the issue of valuation of productivity losses in an entirely different manner. Advocates of the friction cost approach come-up with relatively modest estimates of productivity losses, whereas those using the human capital approach may easily reach estimates that are ten to hundred times higher than those generated by frictionists. There is no easy solution to this problem, and both approaches may hold best arguments, depending on the local circumstances of the analysis. In the meantime, absolute clarity about methodology used to derive productivity costs estimates is indispensable.

Two studies presented the QALY as an outcome. In studies done from a healthcare perspective the QALY as an outcome measure is almost standard practice. Although economic evaluations presenting the QALY as outcome measure are more or less comparable with each other, the way the QALY is measured in a public-health intervention performed from a societal perspective is debatable. The questionnaires to estimate the QALY are directed on health status, while in a societal perspective also other benefits besides healthcare, like social welfare, should be involved; these are not included in those questionnaires [21]. In general, it will be useful to think about the method used to make an economic evaluation of public health programs. Recent literature gives arguments to change from the cost effectiveness/cost-utility analyses to cost-benefit analyses (CBA). CBA does assess how social welfare is affected by an intervention, by identifying and measuring all costs and benefits. All gains, also health gains, are expressed in monetary terms [22]. Clearly this would represent a more holistic approach to evaluation of different alcohol policy measures. However, for obvious reasons, CBA is rare in all economic evaluations, not only for those of public health programmes.

Some limitations of this study have to be taken into account. We calculated per-study averaged total healthcare costs and societal costs, but it is quite difficult to compare costs as reported in different economic evaluations. This was due to differences in study design, outcome measures, discount rates, et cetera. For example, three studies used 'preventing the decrease of drinking' and 'abstinence' as a surrogate outcome measure. Another limitation is that the cost-of-illness studies showed also differences in the types of costs involved in the studies. That means that a full comparison of the studies is not really possible, although these studies are more complete than the economic evaluations. Furthermore, in the original studies, it is not always clear which costs are placed under the types of costs. It will be possible that 'property damage' costs for instance are placed under the topic 'criminal damage'.

## Conclusion

The costs included in economic evaluations on alcohol abuse interventions performed from a societal perspective do not correspond with those included in cost-of-illness studies on alcohol abuse. It is obvious that alcohol abuse not only affects health care costs, but has also a big influence on societal costs. Productivity losses account for the largest proportion of the total costs. Therefore, it is important that all economic evaluations report productivity costs in the same way, to ensure comparability among studies.

Policy makers need to have data based on real life, i.e. all societal costs (next to all health care costs) should be included in economic evaluations from the societal perspective. It can be concluded from the results of this study, that this is not the case in most economic evaluations. In considering whether good value for the money is achieved when introducing a preventive intervention, policy makers should take into account that the result from an economic evolution from the societal perspective might be an underestimation of the true costs and effects of the intervention.

## Competing interests

The authors declare that they have no competing interests.

## Authors' contributions

PFVG and MVDB developed the method of the study. All the authors helped to draft the manuscript, read and approved the final manuscript.
